# Nile Red Incubation Time Before Reading Fluorescence Greatly Influences the Yeast Neutral Lipids Quantification

**DOI:** 10.3389/fmicb.2021.619313

**Published:** 2021-03-04

**Authors:** Mauricio Ramírez-Castrillón, Victoria P. Jaramillo-Garcia, Helio Lopes Barros, João A. Pegas Henriques, Valter Stefani, Patricia Valente

**Affiliations:** ^1^Graduate Program in Cell and Molecular Biology, Biotechnology Center, Universidade Federal do Rio Grande do Sul, Porto Alegre, Brazil; ^2^Research Group in Mycology (GIM), Universidad Santiago de Cali, Santiago de Cali, Colombia; ^3^New Organic Materials and Forensic Chemistry Laboratory (LNMO-QF), Institute of Chemistry, Universidade Federal do Rio Grande do Sul, Porto Alegre, Brazil; ^4^Department of Microbiology, Immunology and Parasitology, Universidade Federal do Rio Grande do Sul, Porto Alegre, Brazil

**Keywords:** Nile red, fluorescence stabilization, lipid quantification, spectrofluorometry, oleaginous yeast

## Abstract

High-throughput screening methodologies to estimate lipid content in oleaginous yeasts use Nile red fluorescence in a given solvent and optimized excitation/emission wavelengths. However, Nile red fluorescence stabilization has been poorly analyzed, and high variability occurs when relative fluorescence is measured immediately or a few minutes after dye addition. The aim of this work was to analyze the fluorescence of Nile red at different incubation times using a variety of solvents and oleaginous/non-oleaginous yeast strains. We showed that fluorescence stabilization occurs between 20 and 30 min, depending on the strain and solvent. Therefore, we suggest that fluorescence measurements should be followed until stabilization, where Relative Fluorescence Units should be considered after stabilization for lipid content estimation.

## Introduction

Nile red (9-diethylamino-5H-benzo[α]phenoxazine-5-one) is one of the most used dyes to visualize lipid droplets and quantify neutral lipids in potential oleaginous microorganisms (Kimura et al., 2004; Sitepu et al., 2014; Balduyck et al., 2015; Alemán-Nava et al., 2016; Rostron and Lawrence, 2017; Deeba et al., 2019; Patel et al., 2019). Different solvents, such as PBS (Phosphate Buffer Solution), DMSO (Dimethyl Sulfoxide), PBS with isopropyl alcohol, or glycerol, are used to facilitate the Nile red penetration inside the cells. However, protocols are not fully standardized, especially concerning the incubation time (time delay between Nile red contact with cells and fluorescence measurement). Kimura et al. (2004) or Zhao et al. (2019), for instance, suggested 5 min of delay on the darkness before the measurement, while Poli et al. (2014a) suggested 10 min. Worries concerning variation on results based on Nile red fluorescence have been reported in the literature: Lamprecht and Benoit (2003); Pick and Rachutin-Zalogin (2012), and Niehus et al. (2018) pointed out that the inexact results of Nile red quantification are due to photobleaching or instability of the molecule. The polarity of the solvent used to dissolve the dye and cells (Chen et al., 2009), and the difficulty for penetration of the dye into cells due to the presence of cell walls (Gao et al., 2008) are also major concerns.

There are few high throughput methodologies for screening oleaginous microorganisms, mainly yeasts. The most used protocols are Kimura et al. (2004), modified by Sitepu et al. (2019), followed by recent publications with modifications proposed by Rostron and Lawrence (2017); Zhao et al. (2019), and Miranda et al. (2020). Our main objective was to evaluate the stability of Nile red fluorescence in different solvents using a protocol based on Sitepu et al. (2019) and a microplate reader equipment. We verified that although the fluorescence peak appears within the first 20 min, variation in the readings among repetitions are high, and fluorescence stability is attained only after 20 min. Therefore, we propose a modification of the protocols by Rostron and Lawrence (2017); Sitepu et al. (2019), and Zhao et al. (2019) to guarantee more robust estimations of lipid content in yeasts.

## Materials and Methods

### Chemical Reagents and Solvents

Nile red (Sigma-Aldrich Co., St. Louis, MO, United States) was dissolved in acetone (100 μg/mL). Different solvents were employed: PBS 1X [137 mM NaCl (Vetec, Brazil), 2.7 mM KCl (Vetec, Brazil), 8 mM Na_2_HPO_4_ (Vetec, Brazil), and 2 mM KH_2_PO_4_ (Vetec, Brazil)], PBS 1X with 5% isopropyl alcohol (v/v), 50% Glycerol (v/v in distilled water), A-gly broth [1 g/L KH_2_PO_4_, 1 g/L (NH_4_)_2_SO_4_ (Cromoline, Brazil), 0.5 g/L M_*g*_Cl_2_-6H_2_O (Nuclear, Brazil), and 15% glycerol (v/v)] and A-gly broth with Dimethyl Sulfoxide (DMSO) 5% (v/v, Sigma-Aldrich Co., St. Louis, MO, United States).

### Yeast Strains and Lipid Accumulation

The oleaginous yeasts *Meyerozyma guilliermondii* BI281A (deposited as UFMG-CM-Y6124 at Microorganisms Collection, Universidade Federal de Minas Gerais, Brazil) and *Yarrowia lipolytica* QU21 (UFMG-CM-Y327) were tested. *Saccharomyces cerevisiae* MRC164 was used as a non-oleaginous yeast. Each strain was grown in YM broth (3 g/L yeast extract, 3 g/L malt extract, 5 g/L peptone, 10 g/L glucose) for 48 h at 28∘C to obtain metabolically active cells. After, we transferred each strain to 25 mL of A-gly broth in a 125 mL flask and grew it for 24 h at 28∘C and 150 rpm. From this pre-culture, we inoculated 1 mL of 7 × 10^7^ cells/mL, estimated by counting using a Neubauer chamber, in 75 mL of A-gly broth in a 250 mL flask (Poli et al., 2014a) for 7 days, 26∘C and 150 rpm on shaker. Previous results obtained by gravimetric and fluorescence approaches showed that *M. guilliermondii* BI281A and *Y. lipolytica* QU21 are oleaginous yeasts (Poli et al., 2013, 2014b; Ramírez-Castrillón et al., 2017).

### Nile Red Stability

Samples containing 150 μL of each solvent, or 150 μL of cells of *M. guilliermondii* BI281A [optical density (OD)_600 *nm*_ = 0.03] suspended in each solvent were transferred to black background flat bottom 96-wells microplates (Jet Biofil, China) without sealing, and the relative fluorescence was measured in a Perkin Elmer Enspire Multimode Plate Reader 2300 equipment (488 nm of excitation, 585 nm of emission, Ramírez-Castrillón et al., 2017). After measuring the basal fluorescence intensity in each well without the fluorescent dye (autofluorescence), we added 50 μL of Nile Red (final concentration: 25 μg/mL) to the solution, shook for 5 min inside the equipment and measured the fluorescence in each well with the dye. The measurement was repeated after 5 min, followed by a kinetic reading every 10 min until 60 min. Each measurement was preceded by shaking for 5 s to suspend the cells. We repeated the experiment using *M. guilliermondii* BI281A (OD_600 *nm*_ = 1) with measurements at 10, 30, 60, and 90 min of incubation. The relative fluorescence expressed as RFU (Relative Fluorescence Units) was obtained after subtraction of both the autofluorescence of the samples and the fluorescence of the solvent in the presence of Nile red (blank).

To evaluate the effect of fluorescence intensity against incubation time in presence of cells (OD_600 *nm*_ = 0.03), independent of the solvent, we constructed a boxplot to visualize confidence intervals, detect outliers and determine statistical differences using the Kruskal-Wallis (non-parametrical) test. This evaluation was repeated with cells with OD = 1 for each solvent. Each sample had technical triplicates. All dataset and detailed data acquisition were described by Ramirez-Castrillon et al. (2020).

### Lipid Extraction and Gravimetrical Determination

To assess the oleaginous character of each strain tested, total lipids, lipid yield (g of total lipids/g of dry biomass), and productivity were determined by gravimetrical methods, according to Ramírez-Castrillón et al. (2017).

### Fluorescence Microscopy

To confirm the staining of Nile red inside cells, representative pictures were taken using optical and epifluorescence modes in a microscope Olympus BX41, according to Storms et al. (2014).

## Results

**FIGURE 1:**
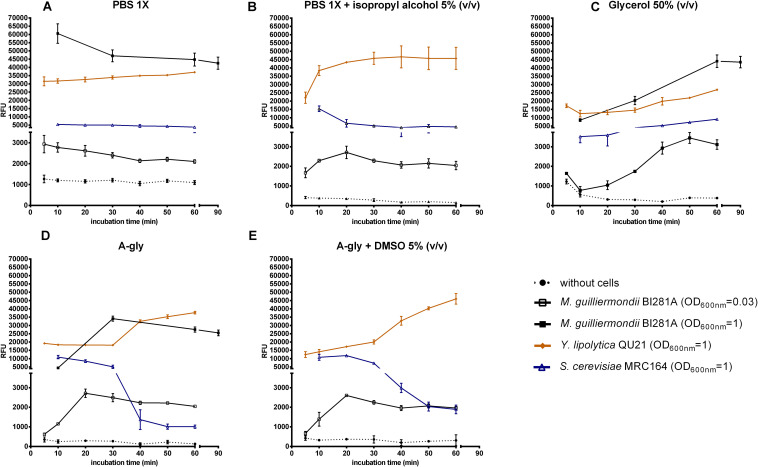
Nile red fluorescence (RFU) against time incubation with the dye for each treatment (min). Treatments included absence (dotted lines) or presence of cells (solid lines). Yeast strains tested: *M. guilliermondii* BI281A (empty square, OD_600 *nm*_ = 0.03; filled square, OD_600 *nm*_ = 1; grown for 7 days in A-Gly broth), *Y. lipolytica* QU21 (diamond, OD_600 *nm*_ = 1; grown for 7 days in A-Gly broth), *S. cerevisiae* MRC164 (triangle, OD_600 *nm*_ = 1; grown for 2 days in YM broth). Solvents: PBS 1X **(A)**, PBS 1X with isopropyl alcohol 5% (v/v) **(B)**, Glycerol 50% (v/v) **(C)**, A-gly broth **(D)**, and A-gly with DMSO 5% (v/v) **(E)**. Y axis was split in two scales (0–4,500 RFU and 5,000–70,000 RFU). Values are means ± standard deviation (*n* = 3). OD, optical density.

Relative Fluorescence Units (RFU) was measured in the absence or presence of oleaginous and non-oleaginous yeast strains. Most treatments without cells showed negligible RFU in comparison with the respective treatment with cells, except for PBS 1X, which presented values higher than 1,000 RFUs (Figure 1A). The analysis of the relative fluorescence of Nile red with the solvent in absence of cells is particularly important, since this measurement is usually applied as the blank in the equipment, and greatly influences on the results.

RFU measurements in the treatments with cells were highly influenced by the solvent used, especially until the first 20 min (Figure 1). The maximum fluorescence with most solvents was achieved in 20 min for *M. guilliermondii* BI281A, *Y. lipolytica* QU21 and *S. cerevisiae* MRC164. As suggested by Sitepu et al. (2012), the maximum fluorescence may be achieved in different times for different yeast strains, and a kinetic reading of the fluorescence until 20 min is necessary for its detection. For PBS 1X (Figure 1A), the fluorescence readings had not a peak, showing a decreasing line with time. However, the behavior of fluorescence changed completely with addition of isopropyl alcohol 5% (v/v) to PBS 1X (Figure 1B), where oleaginous yeasts showed an increasing in fluorescence intensity until 20 min followed by fluorescence stabilization. For *S. cerevisiae* MRC164, the fluorescence intensity decreased until 20 min and stabilized afterward. The behavior in glycerol 50% (v/v) (Figure 1C) was different from other solvents, and the peak was achieved after 50 or 90 min of contact between the cells and the solvent (*M. guilliermondii* BI281A OD = 0.03 and OD = 1, respectively). For *Y. lipolytica* QU21 and *S. cerevisiae* MRC164 we did not observe a maximum fluorescence intensity until 60 min. For A-gly broth (Figures 1D,E), our results did not show differences in absence (Figure 1D) or presence of DMSO 5% (v/v) (Figure 1E). For these solvents, the stabilization was achieved in 20 min for all strains tested. Independent of the optical density of *M. guilliermondii* BI281A, the behavior of these solvents was similar. Also, a lipid accumulation kinetics was constructed for *M. guilliermondii* BI281A with different incubation times of Nile red, and a similar behavior was obtained when PBS 1X, A-gly broth or Glycerol 50% (v/v) were used as solvents (Supplementary Figure S1). In this case, Fluorescence readings were underestimated until 10 min, stabilizing after 30 min.

**FIGURE 2:**
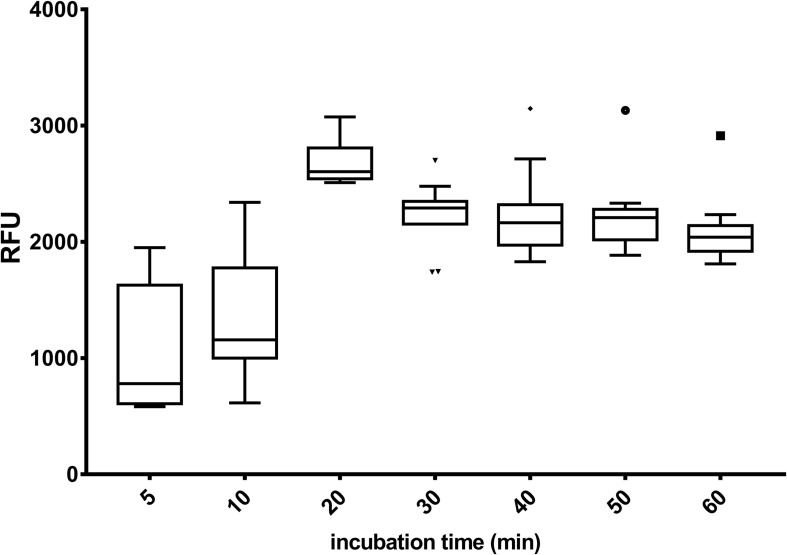
Boxplot of Nile red fluorescence measurements, independent of solvent used, against incubation time. The analysis included cells of *M. guilliermondii* BI281A (OD_600 *nm*_ = 0.03). The box represents 50% of the data, the line inside the box indicates the median (*n* = 3), the confidence interval includes the box and whiskers (95% of the data). Fluorescence readings obtained from glycerol 50% (v/v) were marked as outlier in the boxplot and were not taken into account in the statistical analysis. OD, optical density.

A boxplot was constructed for *M. guilliermondii* BI281A to visualize the confidence intervals of the RFU for each incubation time, independent of the solvent used (Figure 2). The boxplot showed higher widespread fluorescence readings until 20 min of contact between the solvent and the cell (*p* < 0.05). After 20 min, the fluorescence intensity was non-statistically different between incubation times (20 vs. 30 min, 20 vs. 40 min, *p* > 0.05), suggesting stability in the reaction. However, Figure 2 showed that after 30 min of incubation time, the RFU decreased, suggesting a probable photobleaching after 40 min of incubation time. Fluorescence readings obtained from glycerol 50% (v/v) were marked as outlier in the boxplot and were not considered in the statistical analysis. The analysis using a higher cell concentration (*M. guilliermondii* BI281A, OD_600 *nm*_ = 1) was evaluated using four incubation times for the following solvents: PBS 1X, A-Gly broth and Glycerol 50% (v/v). For PBS 1X and A-Gly, the fluorescence was non-statistically different after 30 min (*p* > 0.05), suggesting stability after 30 min of reaction. For Glycerol 50% (v/v), the stability occurred after 60 min of reaction (10 vs. 30 min and 30 vs. 60 min *p* < 0.05; 60 vs. 90 min *p* > 0.05). To confirm the staining with Nile red inside cells, Supplementary Figure S2 shows Nile red staining of *M. guilliermondii* BI281A after growth for 8 days in A-gly broth.

We repeated the same experiment with several yeast strains with or without oleaginous character and we obtained a similar result (Supplementary Figure S3), where stabilization occurred after 20 min, except for *Y. lipolytica* QU21, where we observed an increasing of fluorescence intensity between 30 and 40 min, followed by stabilization. The gravimetric data for these yeast strains is available in Ramirez-Castrillon et al. (2020).

Also, we repeated the lipid accumulation kinetics using different yeast strains and two incubation times with Nile red: 10 and 30 min (Supplementary Figure S4). We measured total lipids and lipid yield for strains BI283, BI296, BI237, and BI282, suggesting that these yeasts are oleaginous (Supplementary Table S1). Comparing the curves of lipid accumulation after 10 min of incubation with Nile red, it seems that BI281A increases the fluorescence until the 8th day of growth. Other strains stabilized lipid accumulation between the 6th and the 8th day, except BI296. However, we suggest that BI296 is still a promising oleaginous strain, with increasing readings of fluorescence (measurements after 30 min of incubation with Nile red) (Supplementary Figure S4B). Other strains, such as BI091, BI231, and BI276, remained with low fluorescence intensities, suggesting they are non-oleaginous yeasts. Other non-oleaginous yeast strains were also measured after 30 min of incubation with Nile red and 5 days of lipid accumulation (data not shown). All data related with these experiments are available in the data repository (Ramirez-Castrillon et al., 2020).

## Discussion

Nile red fluorescence is widely used to screen new oleaginous yeasts (Hicks et al., 2019; Sitepu et al., 2019; Zhao et al., 2019), but also to quantify neutral lipids in non-oleaginous yeasts, such as *S. cerevisiae* or *Schizosaccharomyces pombe* (Rostron et al., 2015). Other dyes are widely used to screen microalgae, such as BODIPY (Rumin et al., 2015), but still with few reports for oleaginous yeasts (for example Patel et al., 2015; Rakicka et al., 2015; Morin et al., 2020). Patel et al. (2019) reported LipidTOX green as a new fluorescent dye to detect oleaginous yeasts. Therefore, few innovations were reported for alternative dyes to stain lipid droplets, so we decided to focus in the most used dye for oleaginous yeasts: Nile red.

Most studies calculate yeast lipid content based on the peak height obtained using a given solvent and optimized excitation and emission wavelengths. Since microorganisms may show the fluorescence peak in different wavelengths, the appliance of high throughput methods for screening of oleaginous microorganisms out of large microbial collections is challenging. Sitepu et al. (2019) proposed following the kinetics of fluorescence until 20 min of contact between the fluorescent dye and the cells, and to choose individual peak heights for lipid content estimation. They suggested using a mixture of the solvent DMSO with the cells in the culture medium plus the Nile red dye and reading the fluorescence kinetics for 20 min with 60 s intervals to detect the fluorescence peak, whose value may be used to calculate the lipid content for the strain. The maximum fluorescence may be achieved in different times for different yeast strains, and a kinetic reading of the fluorescence until 20 min is necessary for its detection. Rostron and Lawrence (2017) proposed a detailed methodology to stain yeast cells with Nile red, however, they suggested fluorescence reading immediately after Nile red addition in the solution and recommended good practices in pipetting and mixing the yeast suspension to reduce variability in the measurements. Zhao et al. (2019) proposed a methodology to stain oleaginous yeasts based in two solvents and established a time range of measurements between 5 and 30 min in darkness, being 5 min the chosen time for the method. On the other hand, Miranda et al. (2020) stated that the maximum RFU values were obtained after 20 min for all tested yeast isolates, supposing that a screening protocol fixing the incubation time is enough to know new oleaginous yeasts. However, these authors did not show the kinetics of lipid accumulation, turning the analysis of Nile red stabilization difficult. Therefore, the fluorescence behavior of Nile red in the presence of any solvent should be carefully evaluated to detect peak fluorescence and stabilization.

Based on our results, we suggest that fluorescence kinetics should be followed until fluorescence stabilization to obtain accurate measurements, and RFU values after stabilization should be considered for lipid content estimation in yeasts, since Nile red fluorescence is almost solvent independent from this point on (Supplementary Figure S1 and Figure 2). Care must be taken in case Glycerol 50% (v/v) if it is used as solvent, since incubation time could be longer (Figure 1). Fluorescence stabilization means that the fluorescent dye passed across the cell membranes into the lipid droplets and attained homeostasis. In the case of all tested strains, stabilization was achieved between 20 and 30 min of incubation, but Nile red fluorescence behavior may be different for other strains (Supplementary Figure S3).

The use of glycerol as facilitator for transport of fluorescent dyes across cell membranes was suggested for microalgae because the cell membrane is permeable to small uncharged polar molecules like glycerol (Rumin et al., 2015), and may be applied for other microorganisms. Glycerol transport across microbial membranes occurs both actively, through a proton symport, and passively, through facilitator channels (Oliveira et al., 2003). The late access of Nile Red to the cell can be related to the different behavior observed with glycerol. Other solvents are known to interact with the cell membrane. For instance, DMSO is known to facilitate the permeation of macromolecules, probably due to induction of water pores across the lipid bilayer and modification of membrane fluidity (Notman et al., 2006). However, we did not find difference when DMSO was added to A-gly, suggesting that addition of this molecule is not necessary.

Another important effect is the sensitivity of the emission spectra of Nile red to the chemical and physical properties of the solvents (Lakowicz, 1983), Nile red concentration (Greenspan and Fowler, 1985), and yeast species (Sitepu et al., 2019), thus excitation and emission wavelengths should also be carefully evaluated to accurately quantify neutral lipids for biotechnological purposes. The wavelengths used in the present study were previously adjusted to allow comparison of the effect of the tested solvents in Nile red. We are aware that the used wavelengths may not be the optima for all the solvents, but the use of a suboptimum wavelength is expected to influence peak height, not overall fluorescence spectrum behavior. Therefore, our results are not intended to quantify yeast lipids but to compare Nile red fluorescence behavior in different solvents.

In conclusion, we warn different researchers, who use Nile red as fluorescent dye to quantify neutral lipids in yeasts, that the proper incubation time after mixing cells with Nile red should be assessed to reach stabilization. Our propose is to mix the solution at least for 20 min, depending on the yeast strain and solvent. The value immediately after fluorescence stabilization should be chosen for accurate yeast lipid quantification. We suggest the use of A-gly broth as solvent. The addition of DMSO to A-gly had no effect on the incubation time or stabilization of readings.

## Data Availability Statement

The datasets presented in this study can be found in online repositories. The names of the repository/repositories and accession number(s) can be found below: http://dx.doi.org/10.17632/8z22js79dk.1.

## Author Contributions

MR-C: conceptualization, investigation, writing—original draft preparation, and visualization. VJ-G: methodology, investigation, and writing—review and editing. HLB: methodology, investigation, validation, and writing—review and editing. JP: resources and supervision. VS: resources, conceptualization, and methodology. PV: project administration, formal analysis, writing—review and editing, and supervision. All authors contributed to the article and approved the submitted version.

## Conflict of Interest

The authors declare that the research was conducted in the absence of any commercial or financial relationships that could be construed as a potential conflict of interest.
