# Supplementary material for: Nile Red Incubation Time Before Reading Fluorescence Greatly Influences the Yeast Neutral Lipids Quantification
Source: Front Microbiol. 2021 Mar 4;12:619313. doi: 10.3389/fmicb.2021.619313 (PMC7969498; doi:10.3389/fmicb.2021.619313)
Supplement: Supplementary file 1 [file Data_Sheet_1.docx]

Supplementary Material

## Supplementary Figures





**Figure S1.** Kinetics of lipid accumulation of M. guilliermondii BI281A grown in A-gly broth for 12 days. Relative Fluorescence of Nile red was measured with PBS 1X (A), A-gly broth (B), or glycerol 50% (v/v) (C) as solvents, and different incubation times. Values are means ± standard deviation (n=3).

**
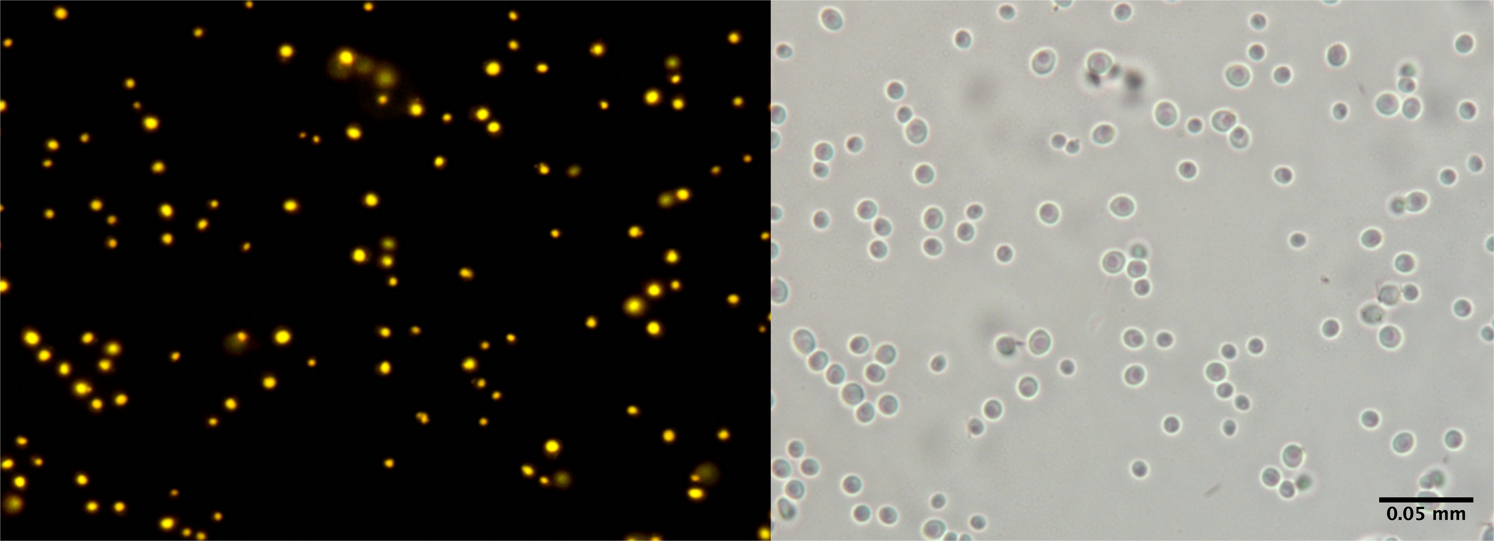
**

**Figure S2**. Nile red staining of *M. guilliermondii* BI281A cells after grown for 8 days in A-gly broth.





**Figure S3.** Nile red fluorescence intensity (expressed as Relative Fluorescence Units) against incubation time with Nile red dissolved in A-gly broth. Yeast strains tested were grown in A-gly broth for 8 days and OD600nm =1. Values are means ± standard deviation (n=3).


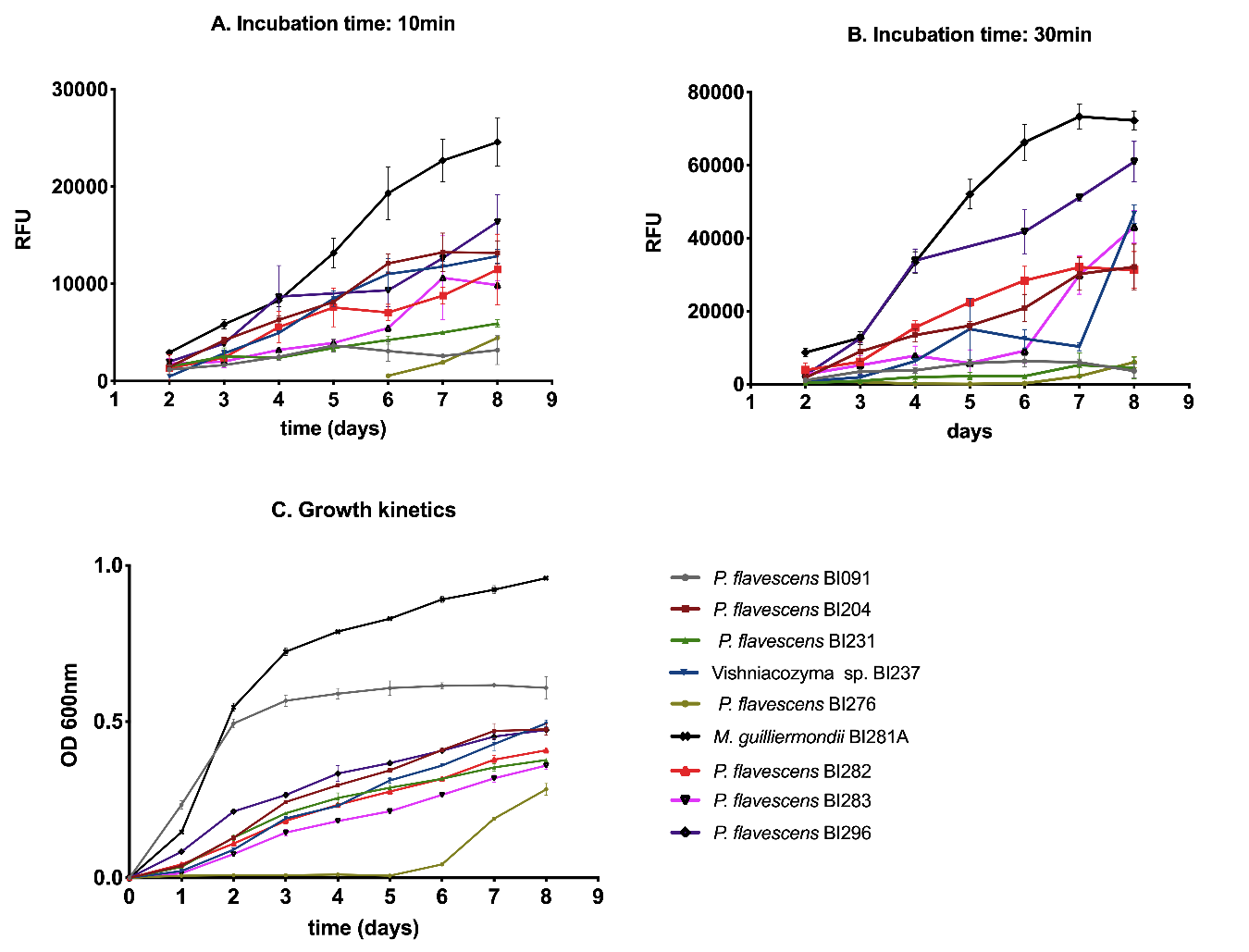


**Figure S4.** Lipid accumulation kinetics for different yeast strains using two incubation times with Nile red: 10min (A) and 30min (B). (C) Growth kinetics using OD600nm. Nile red fluorescence intensity was expressed as Relative Fluorescence Units and was dissolved in A-gly broth. Values are means ± standard deviation (n=3).”

## Supplementary Tables

**Table S1.** Gravimetric analysis for different yeast strains.

| Strain | Replicate | Biomass (g/L) | Total lipids (g/L) | Lipid yield (g/g) |
| --- | --- | --- | --- | --- |
| *P. flavescens* Bi283 | 1 | 5,1760 | 2,0340 | 0,39296754 |
|  | 2 | 4,6980 | 1,1960 | 0,25457642 |
|  | 3 | 2,0296 | 1,1720 | 0,57745369 |
| *Vishniacozyma* sp. Bi237 | 1 | 5,2400 | 1,7460 | 0,33320611 |
|  | 2 | 4,7100 | 1,7640 | 0,37452229 |
|  | 3 | 3,2800 | 1,4560 | 0,44390244 |
| *P. flavescens* Bi296 | 1 | 6,0080 | 1,7740 | 0,29527297 |
|  | 2 | 5,1300 | 1,4380 | 0,28031189 |
|  | 3 | 3,7260 | 1,2460 | 0,33440687 |
| *P. flavescens* Bi282 | 1 | 4,6720 | 1,9880 | 0,4255137 |
|  | 2 | 2,2180 | 1,2640 | 0,56988278 |
